# Efficient downstream processing of second-generation lactic acid from lignocellulosic waste using aqueous two-phase extraction

**DOI:** 10.1186/s40643-025-00847-y

**Published:** 2025-03-19

**Authors:** Irene Gugel, Filippo Marchetti, Stefania Costa, Erika Baldini, Silvia Vertuani, Stefano Manfredini

**Affiliations:** Department of Life Sciences and Biotechnology, University of Ferrara, Via L. Borsari 46, 44121 Ferrara, Italy

**Keywords:** Second-generation lactic acid, Lactic acid extraction/Purification, ATP extraction

## Abstract

The utilization of low-cost, waste-derived lignocellulosic biomass for biotechnological lactic acid production is an area of significant interest. Although numerous attempts have been made to develop novel processes using second-generation feedstocks, the downstream recovery processes for second-generation lactic acid are still relatively uncharted. In this context, this study explores a two-step method for retrieving second-generation lactic acid from a fermented olive leaves medium. The initial step involves an aqueous two-phase extraction using an ethanol/ammonium sulfate system, a method first applied here to lactic acid derived from second-generation raw materials. This is followed by a purification step using activated carbon. The process succeeded in extracting second-generation lactic acid with a yield of 70.02 ± 2.29% in the first extraction stage, followed by a yield of 88.91 ± 0.49% in the second purification step. The lactic acid purity level at the end of the downstream process was 90.17 ± 1.55%, as confirmed by the HPLC data. Furthermore, the study examined the feasibility of direct lactic acid extraction from unfiltered, fermented olive leaves medium, revealing that up to 95.23 ± 0.42% of bacterial cells could be eliminated. The purified lactic acid underwent qualitative identification through FT-IR and ^1^H-NMR analysis, with the spectra showing a high degree of similarity to a standard reference substance, thus demonstrating the use of low-cost waste lignocellulosic biomass for the biotechnological production of lactic acid is a topic of great interest.

**Graphical Abstract:**

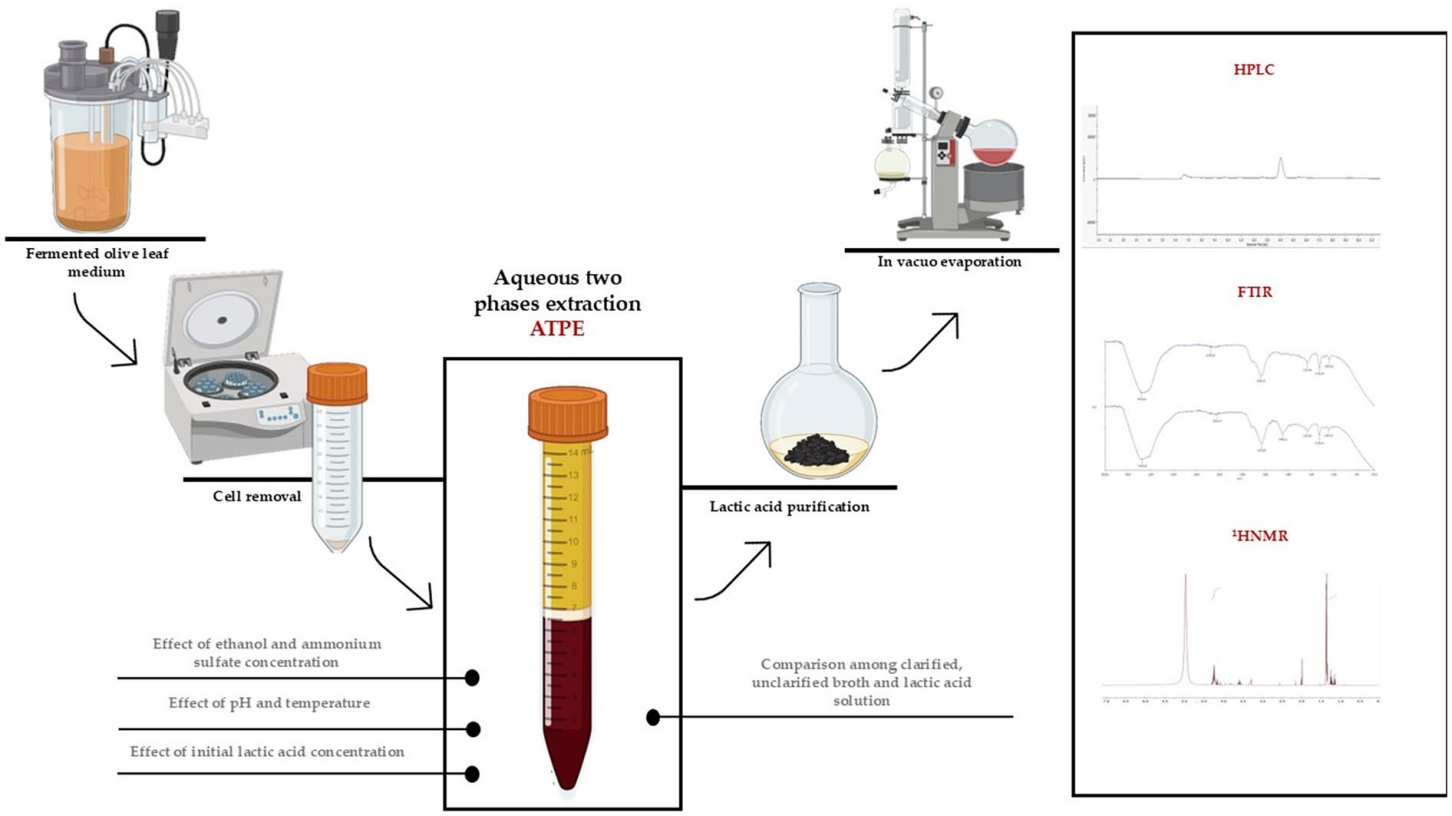

**Supplementary Information:**

The online version contains supplementary material available at 10.1186/s40643-025-00847-y.

## Highlights

● Second generation lactic acid can be recovered by aqueous two-phases extraction.

● ATPE enables high purity 2G-lactic acid to be obtained.

● Performing ATPE, the biomass removal step could be bypassed.

**Supplementary Information:**

The online version contains supplementary material available at 10.1186/s40643-025-00847-y.

## Introduction

Lactic acid (LA), also known as 2-hydroxy-propanoic acid, is a versatile molecule classified under organic acids. Its multifunctional nature allows it to be utilized both in its original form and as a precursor for several other compounds. These include pyruvic acid, ethyl lactate, propylene glycol, and polylactic acid, highlighting the extensive applicability of LA in various chemical and industrial processes (López-Gómez et al. 2020). LA can be produced chemically or biotechnologically. Approximately 90% of LA is now produced via fermentation, which allows for the creation of an enantiomerically pure product with reduced energy use and lower temperatures. However, since synthetic media and refined sugars are too expensive, there has been a growing interest in finding alternative substrates for fermentation (Costa et al. 2020). Lately, particular attention has been paid to low-cost waste lignocellulosic biomass as it is the most abundant source of fermentable sugars available (Ghaffar et al. 2014; de Oliveira et al. 2018; Ojo and de Smidt 2023). The term “second generation lactic acid” (2G-LA) is applied to identify the product of lactic fermentation derived from lignocellulosic waste substrates (de Oliveira et al. 2020).

2G-LA avoids competing with food resources while retaining the benefits of a biotechnological innovation, contributing to social progress by reducing reliance on petrochemicals. Consequently, employing second-generation feedstock over first-generation sources like corn starch is increasingly appealing and preferred because crops that could be used for human consumption are not employed (Alves de Oliveira et al. 2020; Y. Li et al. 2021a, b). In this perspective, some lignocellulosic materials have been studied as substrate for LA production such as sugarcane bagasse and forest and marginal lands biomass residues, but there are needs to evaluate new substrates also to be used as model biomasses (Nalawade et al. 2020; Pontes et al. 2021).

However, although some efforts have been made to find new substrates and develop new processes for the biotechnological production of LA, the downstream process remains a widely discussed topic (Komesu et al. 2017). It is necessary to underline that the downstream process that includes the recovery and purification of microbial LA is not straightforward, especially for the second-generation LA and also depends on the kind of biomass employed (Song et al. 2017; Din et al. 2021). This is largely due to the greater complexity of the fermentation media obtained from 2G-feedstock compared to those derived from first generation feedstock (Yankov 2022). Studies on various downstream processes have been reported in the scientific literature, however, most of these studies have not been performed on 2G-LA obtained from lignocellulosic substrates (Jantasee et al. 2017; Alves De Oliveira et al. 2020). For these reasons, there is an urgent need to identify suitable downstream methods for the recovery and purification of 2G-LA.

The conventional downstream separation process of LA is precipitation (Li et al. 2021a, b). However, Daful et al. pointed out that although this approach is very common, its lack of environmental friendliness risks obscuring the economics of LA produced from lignocellulosic matrices (Daful and Görgens 2017). Adsorption, on the other hand, is a cost-efficient and environmentally less polluting downstream method, but at the same time it has several disadvantages, such as shorter solvent life, poor adsorption capacity and time required (Ahmad et al. 2020). Furthermore, to the best of our knowledge, no studies on this technique applied to 2G-LA are currently available.

Electrodialysis is one of the most promising methods for LA separation and purification, however, a study conducted on LA obtained from coffee mucilage revealed that although a purity of 99.8% can be achieved, the main drawback of this downstream approach is the low recovery rate through the whole process, which was only 38.2% (Joglekar et al. 2006; Neu et al. 2016; Alves De Oliveira et al. 2020; Meng et al. 2020).

Recently, reactive distillation has gained increasing attention for 2G-LA purification (Alves De Oliveira et al. 2020). This latter approach allows simultaneous production and removal of products, improving productivity and selectivity, reducing energy use and eliminating the need for solvents (González-Navarrete et al. 2021). However, as fermented media from second-generation feedstocks are rather complex, the presence of impurities or residual sugars from the fermentation process can adversely affect the reactive distillation process as confirmed by De Oliveira et al. (Alves De Oliveira et al. 2020; de Oliveira et al. 2020).

After evaluating the above-mentioned methods, the salting-out extraction was considered for its low cost, short phase separation time, easy system’s components recovery and easily scalable process. The only disadvantages noted are a low extraction yield of LA from fermentation broths compared to LA standard solutions and a still poor understanding of the mechanisms underlying the extraction mechanism (Xu et al. 2018; Alves De Oliveira et al. 2020).

In conventional salting-out extractions, the system consists of two components: organic solvent, employed as extractant, and inorganic salt, which allows the segregation in two phases when added in a suitable concentration to the solution containing the bio-based molecule. Thus, a salt-rich lower phase and an organic solvent-rich upper phase, in which the target molecule will be parted, are formed (Kumar et al. 2019; Li et al. 2021a, b).

The aqueous two-phase extraction (ATPE) is a variant of salting-out extraction in which the organic phase is hydrophilic. Therefore, ATPE is a biphasic system obtained by mixing two water soluble components with a solution containing the target molecule to be extracted (Dai et al. 2014; Pereira and Coutinho 2020).

ATPE is garnering attention in biotechnology as a straightforward and gentle extraction method. It notably enables the extraction and concentration of different kinds of molecules, typically found in dilute solutions such as fermentation media, into a reduced volume (Hatti-Kaul 2001). This particular type of extraction has already been used for the recovery of lactic acid from whey powder, however it was performed on first generation LA due to the starting matrix used (Aydogan et al. 2011).

Other ATP systems such as ethanol/ammonium sulfate or 2-propanol/ammonium sulfate have been investigated for their potential use as LA downstream process. However, these studies are limited to model solutions (Dai et al. 2014). Song et al. successfully applied the two-step isopropanol/potassium carbonate system for the extraction of 1,3-propanediol and lactic acid from the fermentation of *Klebsiella pneumoniae* in glycerol-based medium (Song et al. 2013). Furthermore, the effectiveness of the ATP system consisting of ethanol and ammonium sulphate has also been demonstrated for other molecules (Li et al. 2010, 2017; Guo et al. 2012).

In consideration of these factors, the current investigation examined ATP extraction as an efficacious method for the downstream recovery of biotechnologically produced LA. Specifically, the study focused on the application of the ethanol-ammonium sulfate system to a fermented LA broth derived from olive leaf waste, representing a novel utilization of this feedstock. The choice of ammonium sulfate as the salting-out agent was made in accordance with the Hofmeister series (Grundl et al. 2017) which indicates that the sulfate anion and ammonium cation are particularly effective in increasing ionic strength. The use of K_2_HPO_4_ as a salting-out agent is also common; however, following the findings of Baral et al. (Baral et al. 2021), it is not the preferred choice since the variables “salt concentration” and “pH” cannot be effectively explored in the extraction of lactic acid from fermentation broth. In fact, K_2_HPO_4_ exceeds the pKa value of lactic acid, and when dissolved in the fermentation broth, it causes a shift in pH towards neutrality. In contrast, the chosen solvent is ethanol, as it is considered the most environmentally friendly according to Byrne et al. (Byrne et al. 2016) in terms of production methods from feedstocks, biodegradability, and the generation of fewer by-products when compared to other water-miscible solvents, such as acetone or THF (Capello et al. 2007).

The objective was to assess the ATP system for processing 2G-LA, which, to the authors’ knowledge, had not been previously investigated. The research evaluated the influence of variables including organic solvent concentration, salt, pH, temperature, and LA concentration in the fermented medium. HPLC analysis was employed for the quantification of both crude and purified 2G-LA, while FT-IR and ^1^H-NMR spectroscopy were utilized for qualitative analysis of the isolated product.

## Materials and methods

### Materials and instruments

Dried olive leaves have been provided from the pruning of an ancient cultivar of *Olea Europaea L.* (Leccino, Moraiolo and Frantoio) grown on the Castello di Monte Vibiano (PG), Italy. pH measurements were performed with Crison Basic 20 pHmeter (Crison instruments, Barcelona). Ammonium sulfate, ethanol, and lactic acid HPLC standard were purchased from Sigma-Aldrich (St. Louis, Missouri, USA). Purified lactic acid collected was characterized by IR with a Spectrum 100 FT-IR spectrometer (Perkin Elmer, Massachusetts, USA) equipped with a personal computer in which the spectra were edited using the Spectrum software and ^1^H-NMR spectrometric analysis using a Varian Mercury Plus 400, operating at 400 MHz. The enantiomeric purity of the isolated and purified lactic acid was tested using a D-/L-Lactic Acid (D-/L-Lactate) (Rapid) Assay Kit (Neogen, Scotland, UK) carrying out the assay following the instructions given by the supplier. The spectrophotometric determination was performed using a UV-6300PC (Avantor VWR, Milano, Italy) spectrophotometer.

### Batch fermentation

The batch lactic acid fermentation process was performed as reported in a previous study (Gugel et al. 2024). Briefly, an olive leaf filtrate was obtained, prepared after a double pretreatment involving hydroethanolic extraction and enzymatic hydrolysis. Subsequently, this filtrate was supplemented with a 10% nutrient enhancer (S4V01) and subjected to lactic fermentation using *Lactobacillus casei*. At the end of the fermentation process, the LA concentration in the fermented olive leaves medium was 28 g/L.

### Phase diagram

The phase diagram identifies the potential working area of an ATP system. For the determination of the binodal curve, the turbidimetric titration method was chosen (Li et al. 2009). Ammonium sulfate at different concentrations was dissolved in 5 mL deionized water and added into a test tube. The slow dropwise addition of ethanol was measured by an analytical balance. After each drop, the mixture was vortexed for 1 min. Ethanol was added until turbid point was reached i.e., when the mixture first became turbid. The ethanol and ammonium sulfate concentrations were calculated at different turbid points and the phase diagram was thus obtained.

### Ethanol/ammonium sulfate aqueous two phases extraction (ATPE)

The fermented olive leaves medium was filtered using 0.45 μm cellulose acetate filters to remove bacterial cells. According to the phase diagram, ammonium sulfate was added to an appropriate quantity of the clarified medium and completely dissolved. Then ethanol was added, reaching a total weight system of 10 g. The mixture was vortexed for 1 min and centrifuged at 6000 rpm for 5 min to facilitate phase separation. In this way a system consisting of an ethanol-rich top phase and an ammonium sulfate-rich bottom phase was obtained. To determine optimal LA extraction parameters, concentration of ethanol was tested in the range 22–30% w/w and ammonium sulfate between 18 and 28% w/w. In addition, the effect of various physicochemical parameters on the partition of LA in ATP systems were investigated. In particular, the influence of temperature in a thermal range of 25–65 °C, the effect of pH (2–6) by adding 10 M HCl, and the initial concentration of LA (28, 60, 100 and 120 g/L) in the fermented medium were studied. Once the best parameters were identified, the results were compared with the unfiltered medium and with a standard LA solution subjected to the same conditions. All the tests were performed in triplicate and the results were expressed as mean value ± RSD%.

The partition of LA in both top and bottom phases was analyzed by HPLC and the partition coefficient (K) [Eq. 1], the phase volume ratio (Vr) [Eq. 2], removal ratio of cells % (Rc%) [Eq. 3] and the extraction yield [Eq. 4] were calculated as follows (Aydogan et al. 2011; Sun et al. 2014).

1 $$\text{K} = \frac{{\left[\text{LA}\right]}_{\text{TOP}}}{{\left[\text{LA}\right]}_{\text{BOTTOM}}}$$

2 $$ \text{Vr} = \frac{{\text{Volume} }_{\text{TOP}}}{{\text{Volume} }_{\text{BOTTOM}}}$$

3 $$ \text{Rc} \text{\%} = (1 - \frac{{\text{C}}_{\text{TOP}}}{\text{Tc}} ) \times  100$$

4 $$ \text{Yield} \text{\%}=\frac{1}{1 + \left[\left(\frac{1}{\text{K} }\right)\left(\frac{1}{\text{Vr}}\right)\right]}\times 100$$

where C_TOP_ and Tc represent the amount of cells in the top phase after ATP extraction and the total cells in the fermented broth before performing ATP extraction respectively.

The ATPE selectivity coefficient of 2G-LA to glucose (denoted as Glu) and that of 2G-LA to lactose (indicated as Lac) were defined as reported by Gu et al. (Gu et al. 2014) [Eqs. 5-6]. To this end, standard solutions of the three analytes were used at a concentration of 100 g/L and under the best extraction conditions determined experimentally.

5 $$ {\text{K}}_{{\text{LA}/}_{\text{Glu}}} = \frac{{\text{K}}_{\text{LA}}}{{\text{K}}_{\text{Glu}}}$$

6 $$ {\text{K}}_{{\text{LA}/}_{\text{Lac}}} = \frac{{\text{K}}_{\text{LA}}}{{\text{K}}_{\text{Lac}}} $$

### Lactic acid purification

Once the best extraction parameters were identified, the purification step was conducted. In order to eliminate as much residual (NH_4_)_2_SO_4_ as possible we followed Xu et al. protocol with slight modifications (Xu et al. 2017). An aliquot of 5 mL LA-enriched ethanol top phase was used and concentrated under vacuum at 30 °C. A twentyfold volume of cold ethanol was added to make the (NH_4_)_2_SO_4_ precipitate. After waiting five minutes at 4 °C, the mixture was centrifuged at 6000 rpm for 5 min for the supernatant recovery. Then, 20% w/v of activated carbon (CA) was added to LA-enriched cold ethanol phase and incubated for 30 min at 25 °C and 130 rpm. Furthermore, the suspension was filtered to recover the ethanolic phase rich in LA which was then placed under vacuum at 30 °C to evaporate ethanol. Subsequently the LA thus obtained was analyzed by HPLC. The CA, on the other hand, was incubated overnight with acetone in a ratio of 1:4 w/v at 30 °C under static conditions (Gao et al. 2011). After the incubation period, the suspension was filtered, and the LA enriched acetone phase was recovered and evaporated under vacuum at 30 °C to remove the acetone. The LA thus obtained was further analyzed by HPLC and characterized via FT-IR and ^1^H-NMR analysis. Moreover, the enantiomeric purity of the purified 2G-LA was also investigated. The purification step was performed in triplicate and the results were expressed as mean value ± RSD%.

### HPLC analysis

The HPLC apparatus consists of Jasco HPLC system (Jasco, Easton, MD, USA) equipped with Jasco RHPLC PU-4180 quaternary pump, Jasco AS-4050 autosampler, Jasco RI-4030 refractive index detector and Jasco UV-4070 UV/Vis detector. Separation was achieved by employing Rezex ROA-Organic Acid H^+^ (8%), 300 × 7.8 mm column (Phenomenex, Torrance, CA, USA) thermostated at 60 °C. A personal computer equipped with chromNAV version 2 software was used for chromatogram acquisition and processing.

Quantitative HPLC analysis was carried out in isocratic condition with a mobile phase made by 0.01 M H_2_SO_4_ in water with a flow rate of 0.6 mL min^−1^. 20 µL of sample was injected after previous centrifugation at 10,000 ×g for 10 min filtration on 0.22 μm cellulose-acetate filter and chromatograms were recorded at 214 nm for the detection of LA; each chromatographic run was completed in 25 min.

Analytical performances of the method were investigated carefully in terms of detection limit, quantification limit, linear range, stability, repeatability, and recovery of the sample.

### AGREEprep assessment

AGREEprep is a framework designed to evaluate the sample preparation phase quickly and efficiently, making it well-suited for the proposed method. AGREEprep allows for the identification of critical points within a protocol, such as those based on extraction methods, before moving on to the analytical phase, and enables researchers to assess in which measure a given protocol aligns with the principles of green analytical chemistry (Pena-Pereira et al. 2022).

## Results and discussion

### Aqueous two phases extraction (ATPE)

#### Phase diagram

In aqueous two-phase extraction, the phase diagram is crucial as it highlights the area where a two-phase system can be obtained, more specifically it allows to identify at which concentrations of salt and organic solvent the creation of two distinct phases occurs. The region where phase separation occurs is above the curve, while below it, the system remains monophasic (Pereira and Coutinho 2020). In this study, the phase diagram plot (Fig. S1) was essential to be able to proceed to the evaluation of the best extraction parameters as well as the partition behavior of LA in ethanol/ammonium sulfate ATP system.

#### Effect of ethanol and ammonium sulfate concentration

**Fig. 1:**
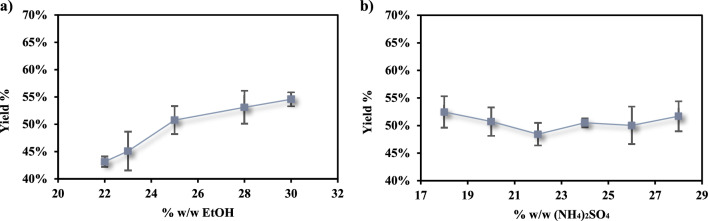
Effect of ethanol (**a**) and ammonium sulfate (**b**) concentration on the LA recovery yield from fermented olive leaves medium

Several factors such as the concentration of the organic solvent and salt, the starting pH of the system, temperature, and initial concentration of the analyte of interest influence the percentage yield of ATPE; therefore, they were considered and explored in this study. The effect of ethanol and ammonium sulfate concentration on the recovery of LA from fermented olive leaves medium is shown in Fig. 1a and b respectively.

Regarding the influence of ethanol concentration on this ATP system (Fig. 1a), an increase from 22 to 25% w/w results in an enhancement of the extraction yield by approximately 8%, whereas subsequent additions lead to a modest upward trend without conferring a significant advantage.

Conversely, the influence exerted by the salt in the ATP system appears to be negligible. Indeed, progressive additions of ammonium sulfate from 18 to 20% w/w do not demonstrate an increasing trend; despite minor fluctuations, the LA extraction yield remains relatively constant. This trend seems to be in contrast with the salting-out effect we want to exploit with this type of extraction. To dissolve a salt, which free anions with high charge density such as SO_4_^2−^ in ATP system, generates a combined effect of both electrostatic repulsion and hydrophobic effect enhancement that, thus, causes the salting-out effect. Due to the entropic penalty, the molecules of interest tend to aggregate and to transfer into the less hydrophilic organic phase (Hyde et al. 2017).

In consideration of these factors, the increase in salt concentration should theoretically enhance the analyte’s recovery. However, in these ammonium sulfate/ethanol aqueous two-phase systems, this phenomenon was not observed. Nonetheless, this particular trend has been previously documented in other studies (Li et al. 2009; Xu et al. 2017).

Xu et al., utilizing an ATP system composed of identical chemical constituents (ethanol and ammonium sulfate) applied to a curcumin solution, hypothesized that the absence of variation in extraction yield, despite the incremental addition of salt, is due to the combined effect of a decrease in phase volume ratio and an increase in top phase polarity (Xu et al. 2017).

Furthermore, it is conceivable that, when working with a complex fermented broth, such as that obtained from olive leaves, even minimal additions of (NH_4_)_2_SO_4_ may contribute to reaching and exceeding the salt’s solubility limit in the solvent. This is also supported by the fact that increasing salt additions have been associated with the formation of a salt precipitate. Therefore, the lack of improvement in the extraction yield could be attributed to the almost complete saturation reached by olive leaves medium after the addition of 18% w/w of salt. Thus, increasing the salt concentration has no advantage in this ATP system.

Due to the results obtained, the concentration of 25% w/w ethanol and 18% w/w ammonium sulfate respectively were chosen for the following experiments.

### Effect of pH and temperature

In aqueous two-phase extractions temperature and pH are described as factors influencing the biomolecules partition. The pH of ATPE may change the charge and surface characteristics of the solute, affecting biomolecule partitioning (Singla and Sit 2023). In fact, if the molecule of interest has a negative charge in water, its partition in the top phase of the ATP system will be favored at higher pH. In the same way, temperature influences biomolecules partition as it determines changes in the ATP system in terms of viscosity and density. However, the way in which the temperature influences the ATP extraction is related to the composition of the system. i.e., the type of organic solvent and inorganic salt that are chosen (Iqbal et al. 2016).

**Fig. 2:**
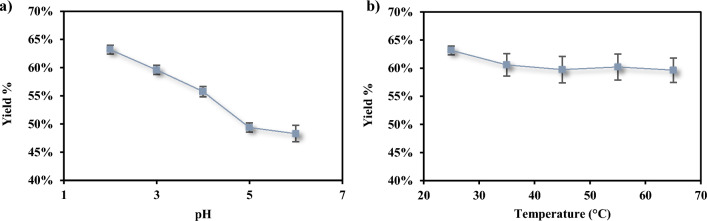
Effect of starting pH (**a**) and temperature (**b**) on the LA recovery yield from fermented olive leaves medium

As can be seen in Fig. 2a, the pH plays an important role in LA recovery. As a matter of fact, increasing the pH from 2 to 6, the extraction yield decreases by about 15% going from 63.16 ± 0.79% to 48.27 ± 1.45%.

Lactic acid (LA) is a weak organic acid with a pKa of 3.86; consequently, the pH influences the extraction yield. When the pH exceeds the pKa, LA predominantly exists in its dissociated form, thereby exhibiting greater affinity for the bottom aqueous phase. Conversely, when the pH is lower than the pKa, LA, being primarily undissociated and thus less polar, becomes insoluble in the bottom aqueous phase and partitions into the top organic phase.

Regarding temperature, no significant effects were observed on the LA extraction yield as shown in Fig. 2b. This observation may be attributed to the brief period required for the aqueous two-phase (ATP) system to achieve equilibrium. Therefore, incubating the system at elevated temperatures proves ineffective, as LA does not further diffuse into the ethanolic upper phase.

A comparable phenomenon was previously reported in a study by Xu et al. In their investigation to optimize the extraction conditions of curcumin in ultrasound-assisted aqueous two-phase extraction (ATPE), they examined the effect of temperature within a range of 20–60 °C and noted that temperature did not significantly impact the extraction yield (Xu et al. 2017).

Thus, in our study, 25 °C and pH 2 were chosen as the best extraction conditions for LA recovery from fermented olive leaves medium.

### Effect of initial lactic acid concentration

**Fig. 3:**
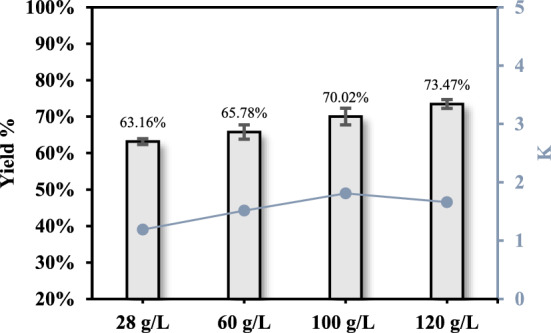
Effect of initial LA concentration in fermented olive leaves medium on the LA recovery yield

Since it was seen that initial LA concentration affects the extraction yield (Hu et al. 2017), four different starting concentrations were used to explore this variable. The ATP extraction was carried out by using the optimum extraction conditions identified in the previous experiments. In the current investigation, a correlation between the starting LA concentration and the extraction yield was found. As can be seen in Fig. 3, there was an improvement from 63.16 ± 0.79% to 73.47 ± 1.22% as the initial LA increased from 28 g/L to 120 g/L. Also, the partition coefficient increased until it reached a plateau. It seemed that increasing the initial LA from 100 to 120 g/L did no longer improve the partition of LA in the upper phase.

Moreover, the concentration of the fermented olive leaves medium presents both advantages and disadvantages. In a more concentrated broth, it is feasible to recover greater quantities of LA utilizing proportionally less organic solvent. Conversely, the concentration process may be economically disadvantageous because, under reduced pressure, it could potentially lead to the polycondensation of LA to oligomer or ester (Hu et al. 2017). For all the above-mentioned reasons, we identified the initial LA concentration of 100 g/L as the most suitable and we did not further investigate more concentrated olive leaves mediums.

#### Comparison among clarified, unclarified broth and lactic acid solution

Using the fermented medium directly for LA extraction, without biomass separation, could decrease processing time and enhance competitiveness. Therefore, the medium, with an initial concentration of 100 g/L, was directly subjected to ATP extraction. The outcomes, as detailed in Table 1, indicate that employing the unclarified broth results in a modest decrease in extraction yield. The extraction coefficient also decreased from 1.81 to 1.60 depending on whether a medium deprived of bacterial biomass was used or not. This decreased partition coefficient could be related to the presence of bacterial cells in the medium that create a kind of clutter that limits the partitioning of LA in the upper organic phase. However, this represents an important issue to be considered. Aqueous two-phase extraction provides a mild environment for the partition and separation of cells. The partitioning of cells is strictly dependent on interfacial tension, the types of salt as well as the bacterial cells used. However, it is generally recognized that an increase in the interfacial tension results in a distribution of cells towards the interface (Cabral 2007; Atefi et al. 2015).

In our study, as reported in Table 1; by using the unclarified broth, it was possible to remove 95.23 ± 0.42% of total cells.

 This result is slightly lower but similar to those reported in other previous scientific studies (Li et al. 2009, 2010; Gu et al. 2014). ATP extraction is also effective when applied to more complex broths containing not only cells but also solid particles. An example is given by Dai et al. who subjected Jerusalem artichoke stem hydrolysate directly to fermentation and ground powder of the tuber was used to feed the fermentation process. In this way, the final fermentation broth had a large amount of solid residue, so that neither centrifugation nor filtration were viable routes. However, using the ATP system consisting of K_2_HPO_4_/ethanol for the extraction of 2,3 butanediol, these solids were precipitated in the lower phase or sequestered in the interphase with a solids removal of 98%. Also in this case, a reduction in the K coefficient of the target molecule was observed in the untreated broth (Dai et al. 2011). The data suggest the promising potential of omitting the biomass separation stage and directly utilizing the unclarified broth for the extraction and concentration of LA via ATP extraction.

**Table 1:** Differences among ethanol/ammonium sulfate systems applied to clarified broth, unclarified broth, and LA solution in terms of yield %, partition coefficient K and removal ratio of cells %

	Clarified broth	Unclarified broth	Lactic acid solution
Yield %	70.02 ± 2.29	68.19 ± 1.61	75.12 ± 1.07
K	1.81	1.60	2.15
Removal ratio of cells %	–	95.23 ± 0.42	–

Regarding this ATP system applied to a 100 g/L LA standard solution, both the extraction yield and the partition coefficient were higher than those of the unclarified and clarified broth. This result was rather predictable since one of the problems of extracting LA from complex fermented broths obtained from 2G-feedstock is precisely the yield gap between them and the simpler LA solution (Alves De Oliveira et al. 2020). In any case, this result is comparable to what has already been reported by applying the ethanol-ammonium sulfate ATP system to a model solution by Aydoğan (Aydogan et al. 2011). Nevertheless, the results obtained with the clarified fermented broth are not so far from those obtained with the LA solution.

Although no substrate remained in our fermented olive leaves medium at the end of fermentation, we calculated the selectivity coefficient of LA on glucose and lactose, which were the substrates of lactic fermentation. In both cases, ATP extraction was more selective for lactic acid than for the two sugars (K_LA/Glu_ = 5.04, K_LA/Lac_= 8.08).

### HPLC analysis

The chromatogram of the fermented medium of olive leaves (Figure S2_A), acquired according to the reported method, shows the peak of LA, whose retention time is 14.1 min, which is accompanied by scattered peaks relating to the presence of other substances in the fermented medium, which are eluted from 7 to 19.5 min.

Instead, from the chromatogram of the ATP extracts from fermented medium (Figure S2_B), it can be easily displayed that the ATP extraction process lowers the collateral peaks but keeps the one related to LA basically unchanged. This underlines the good performance of ATP extraction process, that allows the extraction of most of the LA without co-isolating impurities of the fermented medium.

The chromatogram pertaining to LA, purified under the proposed conditions (Figures S2_C), demonstrates the presence of only the LA peak, exhibiting a substantially unaltered peak area.

The baseline exhibits minimal noise, and the chromatogram is devoid of interfering peaks.

Table 2 presents the results concerning the percentage yield and LA purity for each step of the process.

**Table 2:** Summary data in terms of percentage yield and LA purity of the downstream process steps proposed in the current study

	Fermented olive leaves medium	ATP extract	Purified lactic acid
Yield (%)	–	70.02 ± 2.29	88.91 ± 0.49^a^
LA Purity (%)	52.37 ± 2.86	41.41 ± 0.97	90.17 ± 1.55

^a^The yield of LA in the purification step was calculated by dividing the LA (g) in the ATP extract and the total LA (g) in the purified LA

As far as LA yield is concerned, as mentioned above, 70.02 ± 2.29% of the initial LA could be recovered in the extraction step and 88.91 ± 0.49% in the purification step. Therefore, the overall LA yield was 62.25%. Focusing instead on the purity parameter, fluctuations can be observed. As expected, the purity of the LA within the starting fermented medium is rather low (52.37 ± 2.86%) and this is due to the complexity of the fermented medium. The purity decreases further after the ATP extraction step and this may be due to the fact that this method permits the analytes to be concentrated in a smaller volume than the starting fermented medium. However, as desired, this parameter increased after the last purification step, resulting in a final LA with a purity of 90.17 ± 1.55%. In addition, the purified LA showed a predominance of the enantiomer L (93.15 ± 0.55%).

The most recent studies on lactic acid downstream processes are shown below (Table 3).

**Table 3:** Recent studies on the LA downstream process

Downstream method	LA starting site	Extraction yield	References
Reactive solvent extraction	LA solution	66%	(Erdas and Marti 2024)
Reactive solvent extraction	Fermentation broth (1G-LA)	65%	(Pau et al. 2024)
In-situ DES formation / decomposition	Fermentation broth	99.14%	(Xu et al. 2025)
Adsorption	Fermentation broth	96%	(Wongvitvichot et al. 2023)
Salting-out assisted solvent extraction	Fermentation broth (2G-LA)	85.95%	(Baral et al. 2021)
Solvent extraction	LA solution	22.37% (sesame oil)	(Beg et al. 2022)
Solvent extraction	Fermentation broth (2G-LA)	79.53%	(Alao et al. 2023)
Electrodialysis + Ion Exchange Chromatography	Fermentation broth (2G-LA)	56%	(Azaizeh et al. 2022)
Ion Exchange Chromatography	Fermentation broth	605 mg/g^a^	(Zhou et al. 2023)
Solid-liquid phase microextractive adsorption	Fermentation broth	1150 mg/g^b^	(Zhou et al. 2024)

^a^It refers to the maximum LA adsorption capacity of the IL-335 resin; ^b^It refers to the maximum LA adsorption capacity of the PS-A336 microextractor

Taking into account the most recent studies on 2G-LA, Azaizeh et al. experimented with electrodialysis coupled with ion exchange chromatography to recover lactic acid obtained from the fermentation of carob biomass bagasse after filtration, microfiltration and nanofiltration steps of the fermentation broth. Although it resulted in a lactic acid with a very high degree of purity (99.9%), a considerable amount of LA (46%) was lost during the numerous downstream processing steps. (Azaizeh et al. 2022).

In this study, however, a loss of 37.35% was recorded. An improved result was obtained by Baral et al. who employed a salting-out method for the extraction of 2G-LA obtained from the fermentation of sugarcane bagasse hydrolysate. Specifically, they used ethyl acetate as the organic solvent that is not miscible with water, ammonium sulphate as the salting-out agent and tri-n-butyl phosphate (TBP) as the extractant (Baral et al. 2021).

Regarding the isolation of lactic acid, a performing technique for lactic acid recovery is ion exchange adsorption, a simple, stable, selective, and rapid method. This approach allows for easy scalability, low costs, and the possibility of regenerating the adsorbent resin (Din et al. 2021) with the advantage of integrating lactic acid separation into automated preparative chromatography systems. However, a significant drawback of using ion exchange resins is the large production of effluents resulting from the processing of high feed volumes, a disadvantage that can be minimized if previously ATPE is applied. Implementing ATPE prior to the lactic acid isolation process significantly reduces extract volumes, thereby decreasing the effluent load generated by the process. More sophisticated methods can also be employed for lactic acid purification, such as membrane extraction. This technique ensures high levels of purity and offers flexible systems that can be easily adapted during scale-up. However, the retention of lactic acid and the high cost of membranes are among the main drawbacks associated with this method (Kumar et al. 2019). A green, efficient, and industrially scalable method for lactic acid separation was recently described, involving the in-situ formation of hydrophobic deep eutectic solvents. This method has demonstrated the potential to achieve a 99% extraction efficiency of LA (Xu et al. 2025), and it could be potentially applied to the ATP extracts proposed in this study, offering the advantage of working with smaller volumes, thus contributing to further advancing the system towards a more sustainable frontier.

### Lactic acid characterization

#### FT- IR and 1H-NMR determination of purified lactic acid

A comparative study was performed by acquiring IR spectra of standard solution of LA and LA extracted and purified following the method reported above. From the analysis of the spectra (Figure S2), it is possible to identify a high degree of similarity, particularly referable to the presence of diagnostic bands. First, at high wavenumber values above 3000 cm^−1^ a particularly pronounced broad peak related to the –OH stretching of the acid-carboxylic functionality appears. In the spectral region between 1500 and 2000 cm^−1^ appears the signal related to the stretching/vibration of –C = O carboxylic. At lower wavenumber values, the peak around 1450 cm^−1^ related to the asymmetric/deformation mode of –OH appears in the fingerprint region. In the region from 1200–950 cm^−1^, represented by the C–C and C–O stretching modes, appear a broad peak around 1230 cm^−1^ related to the asymmetric vibration of C-O and around 1130 cm^−1^ a sharp peak related to C–O bonding. Some authors report studies of FT-IR investigation of LA samples, and from the comparison analysis of spectra and characteristic diagnostic peaks, a significant degree of similarity can be identified (Păucean et al. 2017; Huang et al. 2018). ^1^H-NMR spectra of purified LA were acquired after purifying and evaporating under reduced pressure the extract, to reduce any trace of solvent responsible for interference. The LA was then solubilized in deuterated methanol (CD_3_OD) and the spectra were recorded (Figure S3). The diagnostic signals of LA, as can be visualized by analysis of standards and comparison of literature studies (Hu et al. 2017) are at 1.3 ppm with a characteristic doublet type of methyl which forms with methine group of LA an AX_3_ spin system (Govindaraju et al. 2000). The second diagnostic signal detected in the spectrum corresponds to a quadruplet signal at 4.09 ppm, related to the methine signal. By analyzing the spectra of LA obtained by fermentation, isolated, and purified according to the proposed method, the presence of the doublet signal at 1.35 ppm related to methyl and the quadruplet signal at 4.15 ppm diagnostic of the methine substituent can be observed. In addition, it is possible to display at 3.3 ppm the characteristic signal of the analysis solvent CD_3_OD and at 5 ppm the characteristic signal of H_2_O. Scattered peaks are also present in the area between 2 ppm and 4 ppm, probably related to the presence of a minimal amount of impurities in the sample, basically characterized by an almost unrecognizable integral of the peak.

#### AGREEprep assessment

AGREEprep was utilized to identify the main critical points of the method in accordance with green chemistry principles. Nowadays, it is increasingly important to conduct an assessment that evaluates the sustainability of different phases of the process. This approach helps determine the potential environmental, economic, and professional impacts of the protocol, ensuring that the method aligns with the goals of reducing ecological footprint, optimizing costs, and adhering to sustainable practices. This tool employs a color-coded scale ranging from red to green to indicate the sustainability impact associated with each component of the evaluated process. The results are displayed in a graphical representation where the overall score, derived from the integration of scores assigned to each evaluated aspect, is shown at the center. The overall score (placed in the center of the pictogram) approaching a value of 1 indicates highly sustainable processes and are typically represented by the color green. Conversely, scores nearing 0 suggest processes with significant sustainability challenges and are usually marked in red (Pena-Pereira et al. 2022). Through this scoring system and color scale, AGREEprep facilitates a quick and effective evaluation of the sample preparation process, enabling users to immediately identify areas that may require improvement.

**Fig. 4:**
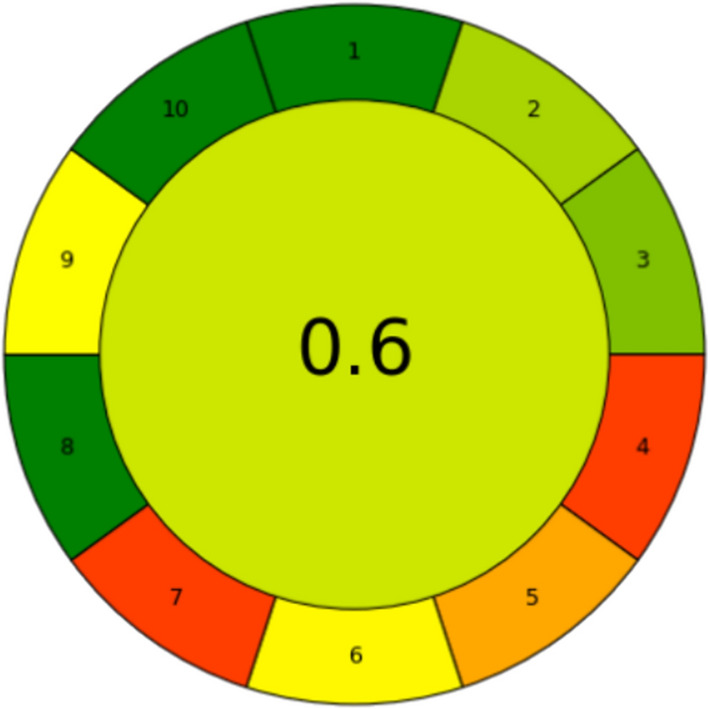
AGREEprep assessment of ATPE in selected extraction conditions. Numbers in the corollary are representative of 1—sample preparation placement, 2—hazardous material, 3—sustainability, renewability, and reusability materials, 4—waste, 5—size economy of the sample, 6—sample throughput, 7—integration and automation, 8—energy consumption, 9—post-sample configuration for the analysis, 10—operator’s safety

As reported in Fig. 4, the primary issues identified by AGREEprep in the ATPE method for LA isolation are reflected in points 4, 5, and 7, which correspond to waste generation, sample size economy, and integration and automation, respectively. According to AGREEprep, minimizing waste production is crucial, aligning with principle 5, which concerns sample size to be as small as possible to enhance the process’s sustainability profile. It is important to note that the sample quantity used in this method was the absolute minimum required to process enough substance for the analytical determinations performed. Moreover, the by-products generated are water-based and do not contain chemicals considered toxicologically significant for either the environment or the operators. The final critical issue affecting the proposed method refers to point 7, which evaluates the degree of process automation. The central value, calculated by the software, corresponds to the overall impact of the greenness of each contribution evaluated in the assessment. In the presented study, the steps involved in the isolation of lactic acid such as the addition of extracting agents, mixing, and separation were conducted manually. However, it is crucial to automate the entire process as much as possible, particularly with an eye toward scalability and industrial implementation. Assessing the potential for scaling up downstream processes to an industrial degree becomes a key factor for the practical application of the method. Alongside this, conducting a techno-economic analysis is essential to provide a realistic forecast of the costs associated with the entire process. Therefore, scaling up represents a fundamental objective for future research goals, with the current study laying a promising foundation for such advancements.

## Conclusions

The experimental work presented here has made significant strides in downstream processing of 2G-LA from lignocellulosic waste, an abundant and renewable resource that does not compete with food supplies. In detail, the study ventured into two-step aqueous extraction (ATPE) as a viable downstream process. It is a gentle extraction technique that concentrates biotechnological molecules from dilute solutions into smaller volumes, offering a potentially scalable alternative for LA recovery. This method, notably used for the first time with an ethanol-ammonium sulfate system on fermented broth from olive leaves, proved effective.

The optimal parameters for ATP extraction were identified, including solvent and salt concentration, pH, and temperature. These parameters were optimized obtaining a yield of recovered LA of 70.02 ± 2.29%. High-performance liquid chromatography (HPLC) and spectrometric analysis (FT-IR and ^1^H-NMR) were used to quantify and qualitatively evaluate crude and purified 2G-LA, demonstrating the accuracy and reliability of the method. Significantly, the study provides data supporting the potential to bypass the biomass removal step by directly using unclarified fermented broth for LA extraction, which could streamline the process and increase its competitiveness despite a slight reduction in yield. The final product achieved a high degree of purity (90.17 ± 1.55%), strengthening the effectiveness of the developed ATP extraction process.

In essence, this research paves the way for a new downstream approach for 2G-LA recovery by trying to fill the lack of studies concerning the extraction and purification of microbial LA from lignocellulosic matrices.

## Supplementary Information

Supplementary material 1.

## Data Availability

All data generated or analysed during this study are included in this published article and its supplementary information files.
