# Supplementary material for: Efficient downstream processing of second-generation lactic acid from lignocellulosic waste using aqueous two-phase extraction
Source: Bioresour Bioprocess. 2025 Mar 19;12(1):20. doi: 10.1186/s40643-025-00847-y (PMC11923332; doi:10.1186/s40643-025-00847-y)
Supplement: Supplementary file 1 — Supplementary material 1. [file 40643_2025_847_MOESM1_ESM.docx]

Supplementary Material

**Efficient Downstream Processing of Second-Generation Lactic Acid from Lignocellulosic Waste Using Aqueous Two-Phase Extraction**

**Irene Gugel^1†^, Filippo Marchetti^1†^, Stefania Costa^1,2*^, Erika Baldini^1^, Silvia Vertuani^1^, Stefano Manfredini^1^**

^1^ Department of Life Sciences and Biotechnology, University of Ferrara, Via L. Borsari 46, Ferrara, 44121, Italy; irene.gugel@unife.it (I.G), filippo.marchetti@unife.it (F.M.), erika.baldini@unife.it (E.B), stefania.costa@unife.it (S.C.), silvia.vertuani@unife.it (S.V), smanfred@unife.it (S.M.)

^2^ Department of Chemical, Pharmaceutical and Agricultural Sciences, University of Ferrara, Via L. Borsari 46, Ferrara, 44121, Italy. stefania.costa@unife.it (S.C.)

^*^ Corresponding author.

†Irene Gugel and Filippo Marchetti equally contributed to this work

**Figure S1.** Phase diagram of ethanol/ammonium sulfate ATP system


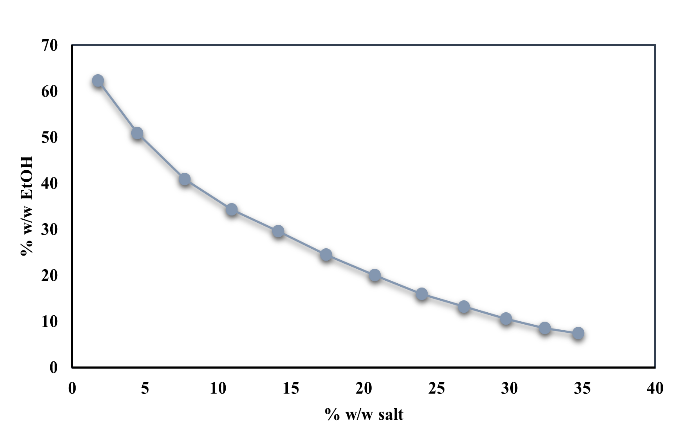


**Figure S2.** Chromatogram of the fermented olive leaves medium (A) Chromatogram of ATP extracted lactic acid (B) Chromatogram of purified lactic acid (C). Caption refers to chromatographic performances of the method.

A)

*
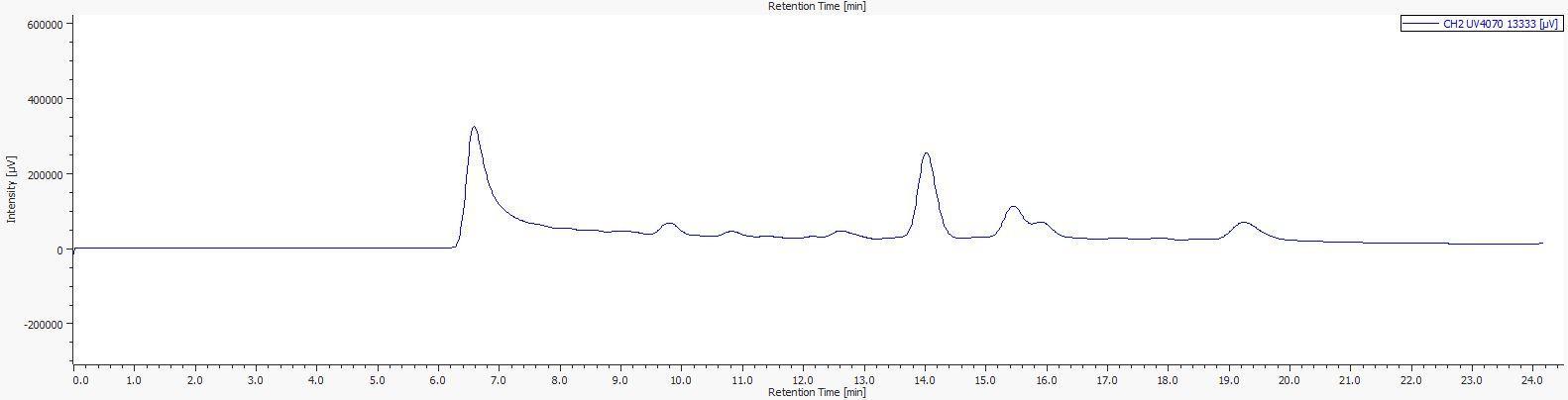
*
B)


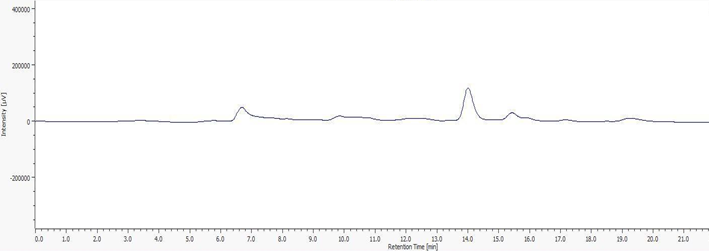


C)


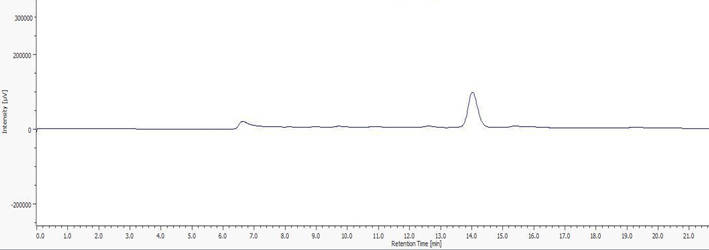


The different samples of LA extracts were quantified with HPLC. before, the analytical method was carefully evaluated and established before measurements. Calibration curves were obtained by injecting LA standards in the range of 1-50 g*L^-1^, reporting peak area values against concentrations. Correlation coefficients were calculated and showed excellent linearity in the range 1-50 g*L^-1^ with R^2^ values greater than 0.999. Limits of detection (LOD) and limits of quantification (LOQ) were calculated by referring to standard deviation intercept method, by the equations below:

$$LOD=3,3\sigma/S$$

$$LOQ=10\sigma/S$$

where S is the slope of the calibration curve and σ is the standard deviation of the response calculated from the calibration curve. LOD value was 0,001g*L^-1^ and LOQ 0,004 g*L^-1^. The reliability of the proposed method was evaluated through interday and intraday precision of triplicate measurements of standard solutions of LA. The results obtained from the stability studies were expressed as the Relative Standard Deviation percentage (RSD%) of the triplicates referring to the peak area of standards. RSD% values referring to intraday precision studies were in the range of 0.5-1.18 while in the interday precision studies RSD% were in the range 1.33-3.78%. From the analysis of RSD% for both the intraday and interday precision, values prove to be well below 5%, confirming that the proposed method is markedly stable. Repeatability studies were performed by injecting three different ATP purified extracts obtained by the optimized method in triplicate. The values inherent to peak area in repeatability studies were expressed as RSD% and showed values in the range of 0.68-1.69%, demonstrating that the proposed method is repeatable. Finally, recovery studies were performed to investigate the accuracy of the proposed method. The purified extracts were spiked at three different levels of excess standards, corresponding to 50%, 100%, 150% LA, respectively. Recovery tests were repeated three times and each sample injected in triplicate. The RSD% values associated with the experimental recovery values are in the range of 1.84-3.29%, while the percent recoveries from the theoretical ones are shown to be 98.85% for 50% analytical excess, while they were 97.82% and 93.37% for standard excesses of 100% and 150%. Accuracy studies yielded RSD% less than 5%, demonstrating a good stability of the proposed method and its suitability in the quantitative determination of LA in purified LA extracts.

Through injection of the fermented medium, ATP extract and purified extract, it was shown that, after 20 minutes of elution, no other peaks related to substances retained in the column were observed. Therefore, subsequent analyses were performed with a run time of 25 minutes and acquisition time was set at 24.5 minutes.

Under the optimized conditions, chromatograms of the samples under analysis were acquired.

**Figure S3.** FT-IR of purified LA (blue line) and standard LA (black line)


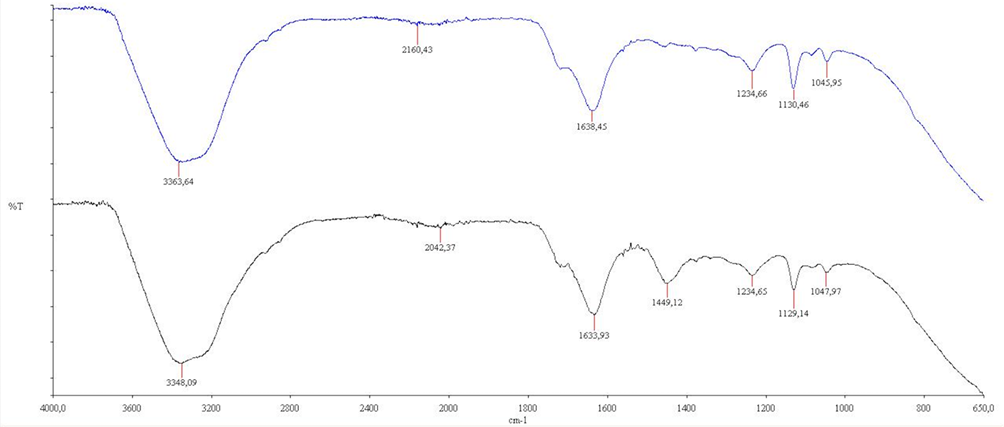


**Figure S4.** ^1^H-NMR of purified LA in CD_3_OD

*
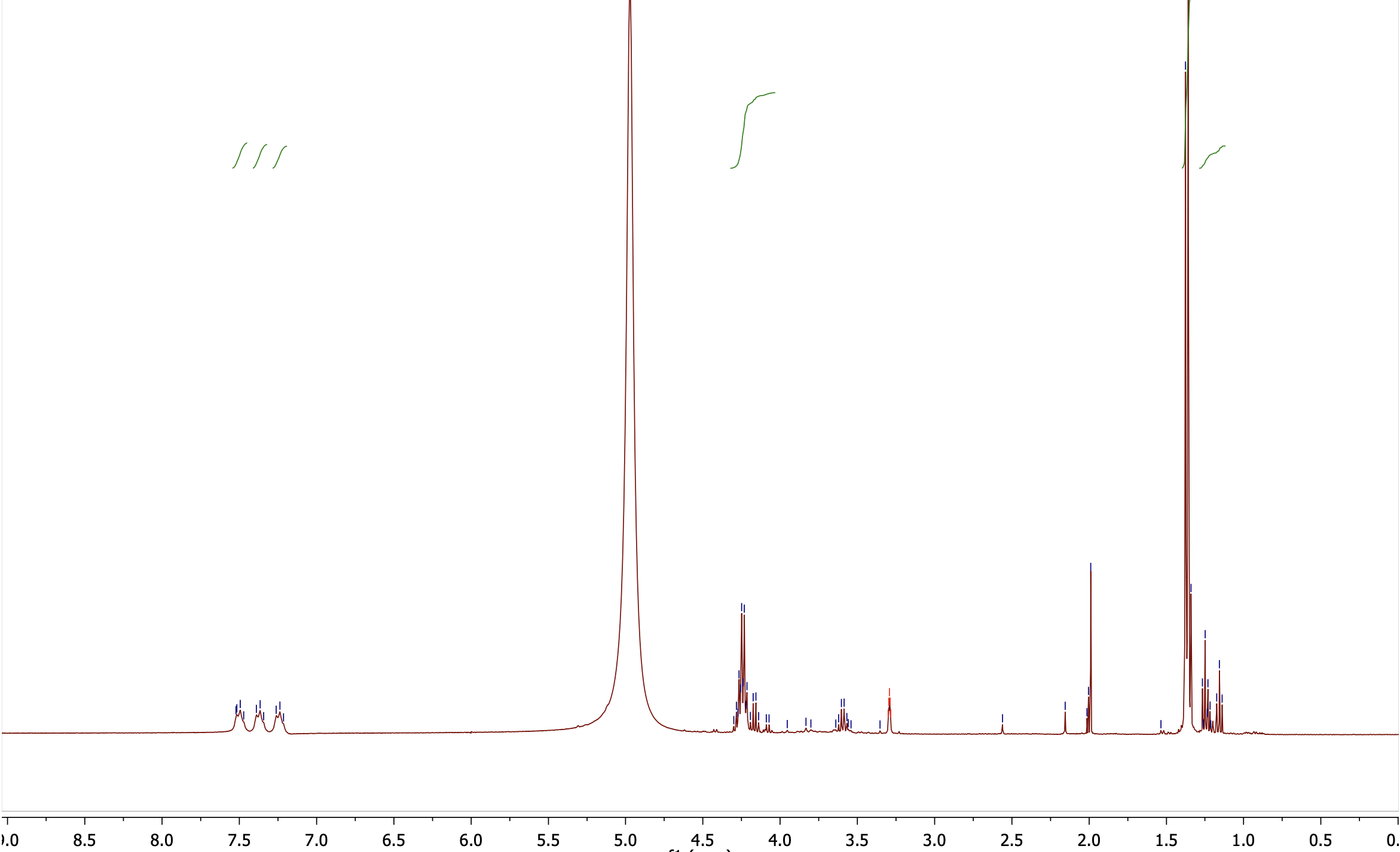
*
